# Persisters—as elusive as ever

**DOI:** 10.1007/s00253-016-7648-8

**Published:** 2016-06-04

**Authors:** Niilo Kaldalu, Vasili Hauryliuk, Tanel Tenson

**Affiliations:** University of Tartu, Institute of Technology, Nooruse 1, 50411 Tartu, Estonia; Department of Molecular Biology, Umeå University, Building 6K, 6L University Hospital Area, SE-901 87 Umeå, Sweden; Laboratory for Molecular Infection Medicine Sweden (MIMS), Umeå University, Building 6K and 6L, University Hospital Area, SE-901 87 Umeå, Sweden

**Keywords:** Antibiotic tolerance, Antibiotic resistance, Persisters, Bet hedging, Antibiotic development, Bacterial infections

## Abstract

Persisters—a drug-tolerant sub-population in an isogenic bacterial culture—have been featured throughout the last decade due to their important role in recurrent bacterial infections. Numerous investigations detail the mechanisms responsible for the formation of persisters and suggest exciting strategies for their eradication. In this review, we argue that the very term “persistence” is currently used to describe a large and heterogeneous set of physiological phenomena that are functions of bacterial species, strains, growth conditions, and antibiotics used in the experiments. We caution against the oversimplification of the mechanisms of persistence and urge for a more rigorous validation of the applicability of these mechanisms in each case.

## Introduction

Development of antibiotics is one of the major achievements of medicine. Unfortunately, this class of drugs is undermined by bacterial evolution that leads to development of mechanisms that reduce their efficacy. One of these mechanisms is antibiotic resistance, the ability of a microorganism to grow in the presence of increasing concentrations of a drug. Resistance is inheritable and relies on specific genes or mutations, such as genetic changes responsible for modification of drug binding sites, antibiotic efflux by pumps, modification of the drug molecule, and others (reviewed by Palmer and Kishony 2013; Wilson 2014; Blair et al. 2014). Another strategy employed by bacteria to counter antibiotics is tolerance, i.e., avoiding death by antibiotics. In contrast to the resistant bacteria, tolerant bacteria are unable to multiply in the presence of a drug but survive the antibiotic challenge. A special case of this strategy is the formation of a subset of extremely tolerant “persister cells.” In an isogenic antibiotic-sensitive culture, persisters constitute a sub-population that survives the treatment by bactericidal antibiotics without harboring a specific antibiotic resistance determinant (Lewis 2010; Balaban 2011). We argue against approaching the phenomenon of persistence by applying concepts that are borrowed directly from the field of antibiotic resistance, i.e., attempting to reduce the persistence to a single defined trait in a sub-population that is brought about by specific molecular mechanisms. In so doing, the research community has reached a greatly oversimplified view of persistence. We argue that the phenomenon of persisters is more complex and that there is no overarching “general mechanism” that can be applied to all bacterial species, regardless of the growth conditions or the class of antibiotic employed.

As a discussion paper, this review touches on only some of the critical points in the field and does not provide a comprehensive overview. For those who are new to this field, we recommend the excellent reviews by Lewis (2010), Balaban (2011), Cohen et al. (2013), Maisonneuve and Gerdes (2014), Amato et al. (2014), and Brauner et al. (2016).

## Persisters, bacterial non-replicating state, and antibiotic tolerance

Bigger (1944) made the astute observation that penicillin cannot sterilize bacterial cultures. The surviving bacteria, the “persisters,” cannot grow in the presence of the drug but resume growth after removal of the antibiotic. In this way, the persisters differ from resistant cells that can grow in the presence of the antibiotic (Lewis 2010; Balaban 2011). Because the cultures started by the surviving cells contain persisters at the same frequency as the original cultures, we know that persistence is not genetically inheritable but is due to phenotypic diversification (Bigger 1944; Keren et al. 2004a). Bigger’s seminal work put forward an idea that persisters are refractory to killing because they are not proliferating; it had already been established that penicillin does not kill non-growing cultures (Meyer et al. 1942). The attempts to solve the riddle of antibiotic persistence in the interim have been scarce (e.g., Moyed and Bertrand 1983) in spite of the suggested clinical importance of this topic (McDermott 1958). Revival of the interest in persisters in 2000s started from a study that suggested a link between survival of a tolerant sub-population and bacterial biofilm resistance to antimicrobials (Lewis 2001; Spoering and Lewis 2001). Newer techniques of single cell analysis (microfluidic devices and flow cytometry) clearly demonstrated the presence of a non-growing sub-population within a growing bacterial culture and that the persisters originate from among these non-growing bacteria (Balaban et al. 2004; Roostalu et al. 2008; Jõers et al. 2010). These observations substantiated the hypothesis that persisters are a minor sub-population of bacteria that maintain a non-replicating state in conditions where most cells are replicating.

However, not all the non-replicating cells resume growth and form colonies upon plating that is needed to define or classify them as persisters. The difference in the size of the non-growing and persister populations can be several orders of magnitude. For example, in conditions where *E. coli* cultures contain <0.2 % persisters, as determined by counting *c*olony *f*orming *u*nits (CFU), ∼20 % are non-replicating and not lysed by ampicillin (Roostalu et al. 2008). These non-replicating and non-lyzing cells have been physically isolated to analyze their molecular content (Cañas-Duarte et al. 2014; Keren et al. 2004b; Keren et al. 2011), although interpolation of these results onto the “real” persisters that constitute a tiny fraction of the non-lyzed bacteria that can be identified only after plating remains speculative. This is also true when a sub-population of cells is physically separated, based on some other marker of inactivity or dormancy (Shah et al. 2006). Most of the non-replicating and non-lyzed cells can be identified as alive using marker dyes that penetrate only dead cells (Keren et al. 2004b; Roostalu et al. 2008).

In environmental microbiology, non-growing bacteria that cannot form colonies are referred to as *v*iable *b*ut *n*ot *c*ulturable (VBNC). They are non-replicating but, in contrast to the dead microorganisms, retain cell integrity, which is typically assessed by impermeability to certain fluorescent dyes, and some degree of metabolism, usually determined with metabolic marker dyes (Pinto et al. 2015; Xu et al. 1982; Oliver 2010). In environmental bacterial communities, VBNC bacteria form the majority: only 0.001–1 % of all microbes can be cultivated on solid media (Bianchi and Giuliano 1996). On one hand, non-cultivability of environmental bacteria is a characteristic of certain bacterial species and entire phyla that cannot be cultured by standard plating methods (Rappé and Giovannoni 2003). On the other hand, it is a phenotypic trait of individual bacterial cells of cultivable pathogens and, as such, has relevance to public health, food technology, and wastewater treatment (Desmonts et al. 1990; Oliver 2010; Li et al. 2014; Ayrapetyan et al. 2015a). The term has been also used to describe bacteria that survive wastewater treatment processes such as chlorination. Thus, the non-replicating sub-population encompasses both VBNC cells and persisters (Li et al. 2014; Ayrapetyan et al. 2015a), which have rarely been discussed in the same context, although they could form a “dormancy continuum” (Ayrapetyan et al. 2015b).

**Fig. 1:**
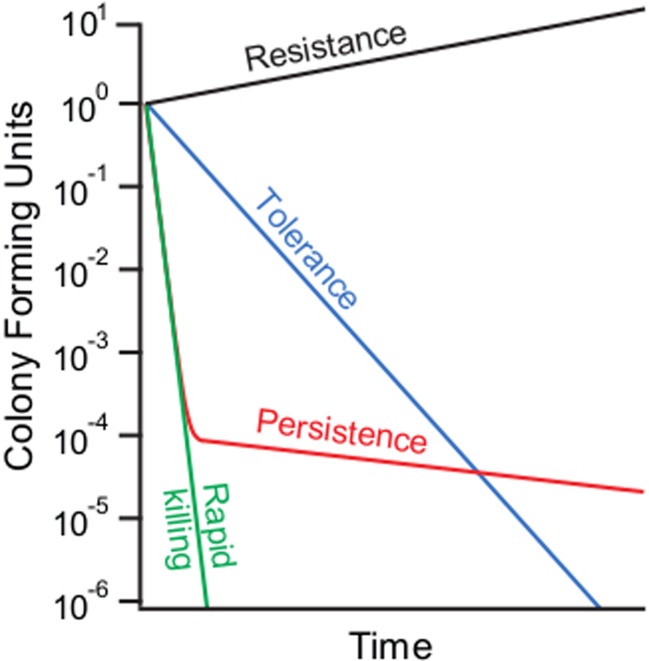
Antibiotic killing kinetics of bacterial cultures showing a rapidly sterilized culture, tolerance and resistance. The slower killing phase or plateau is defined as persisters

Another relevant matter is antibiotic tolerance. Confusingly, antibiotic tolerance and persistence are often used as synonyms; indeed, both characterize bacteria that survive the antibiotic challenge (Kester and Fortune 2014). However, “antibiotic tolerance,” as used in clinical microbiology, describes reduction in the rate of antibiotic-induced killing of a whole bacterial population (Tomasz et al. 1970; Tuomanen et al. 1986a), whereas persistence refers to survival of a small sub-population (Fig. 1). The two phenomena can be distinguished experimentally by following the time course of antibiotic killing, which often has a pronounced biphasic nature. During the first phase, the bulk of the population is killed, followed by a slower phase (or a plateau) corresponding to the persister fraction. Antibiotic tolerance manifests in slower killing during the first phase, whereas increased persistence in *hi*gh *p*ersistence (*hip*) mutants is reflected in a higher plateau. However, the trajectories of the killing curves are often more complex than depicted in Fig. 1 (Lechner et al. 2012), indicating physiological heterogeneity within persister sub-populations (Allison et al. 2011a). Persisters can survive antibiotic concentrations considerably above the minimum inhibitory concentration (MIC). Like the plateau on a *time-dependent* killing curve (Fig. 1), a plateau forms on a *concentration-dependent* killing curve, indicating the range of antibiotic concentrations that kills all non-persisters but allows only the persisters to survive. Analyses of persisters use concentrations of antibiotics within the range of the plateau. Therefore, survival of persisters does not strictly depend on the antibiotic concentration, whereas the killing rate of the bulk of bacteria is dependent on antibiotic concentration and tolerance of the bulk is tested at significantly lower antimicrobial concentrations (Tuomanen et al. 1986a; Theodore et al. 2013).

Antibiotic tolerance can be either genotypic or phenotypic. Genotypic tolerance characterizes clinical isolates and mutants killed at a reduced rate in culture conditions where standard strains are effectively killed. Phenotypic tolerance occurs under cultivation conditions resulting in slow bacterial growth (Meyer et al. 1942; Tuomanen et al. 1986a). For example, the rate of killing by cell wall synthesis inhibiting antibiotics is negatively correlated with bacterial growth rate (Tuomanen et al. 1986a); starving, dormant, non-growing bacterial cultures are tolerant to nearly all antibiotic classes (Tuomanen et al. 1986b). Thus, like the “dormancy continuum,” there is also a “tolerance continuum” of microbes having different genotypes and physiological states. This makes direct comparison of data obtained by different research groups in different conditions exceedingly challenging. In order to address this issue, Brauner and colleagues have put forward a classification of different aspects of tolerance and persistence in a recent Opinion article (Brauner et al. 2016).

## Dormancy and activity of persisters

Connections between the physiological states of bacteria (including persisters) during infection and antibiotic treatment efficiency are not well characterized, but the evidence of such a connection is increasing. Since persisters are believed to be a major cause of the refractoriness of chronic infections, it is natural to discuss them in a medical context and investigate this phenomenon in eukaryotic cell cultures and in the relevant infection models. In these more complex setups, there is greater heterogeneity than in test tube cultures. It is noteworthy that, for *Mycobacterium* infections in which persisters form a highly clinically relevant population responsible for the long duration of antibiotic treatment, non-replicating and actively replicating bacteria (i.e., phenotypically resistant bacteria) coexist (Adams et al. 2011). It is often stated that persisters are dormant cells with diminished metabolism. This notion equates non-replicating state with dormancy and low metabolic activity. While a fraction of mycobacterial persisters is, indeed, metabolically dormant, there is also a fraction of cells that are physiologically active and have extremely efficient drug efflux which is responsible for their survival of antibiotic treatment (Adams et al. 2011; Wakamoto et al. 2013). A connection between the efflux activity and persistence has recently been reported for *E. coli* (Pu et al. 2016).

In an animal model, *Salmonella* forms sub-populations with a wide range of growth rates (Helaine et al. 2014; Claudi et al. 2014). Dormant cells survive antibiotic treatment best, with the fastest growing cells being the most sensitive (Claudi et al. 2014). Dormant cells, although refractory to antibiotics, are not the major contributors to the progression of infection because they do not divide. The slowly dividing cells were the major contributors to the progression of infection because they are less sensitive than the fast growers. This suggests that, during an infection, we should not classify bacterial cells into persisters and non-persisters because a wide range of growth rates and antibiotic sensitivity levels develop.

## Different types of persisters—are persisters equally recalcitrant to all classes of antibiotics?

Many investigations show that this is not the case. Early work showed that *hip* mutants selected using ampicillin had increased persister levels against fluoroquinolone antibiotics, and vice-versa (Wolfson et al. 1990), which suggested that persisters are recalcitrant to several or all antibiotics. However, later studies indicate that persister levels depend considerably on the class of antibiotic and different (probably overlapping) cell populations survive different antibiotics (Goneau et al. 2014; Amato and Brynildsen 2015). For example, bacteria in log phase cultures that are not killed by fluoroquinolones and cell wall synthesis inhibitors were effectively killed by aminoglycosides (Allison et al. 2011b; Jõers et al. 2010; Spoering and Lewis 2001). The underlying mechanism is well understood; aminoglycoside killing depends on membrane potential, which is required for the uptake of the positively charged drugs; stimulation of the potential by metabolites greatly enhances aminoglycoside killing (Allison et al. 2011b). Another convincing example of the differential effects of different antibiotic classes on persister killing was presented in a recent work where a culture was co-treated with ampicillin and ofloxacin before and after a diauxic shift (Amato and Brynildsen 2015). Before diauxie, the persister levels were comparable in co-treatment and separate treatments. After the metabolic shift, which induced persister formation, the persister population of the co-treated sample was tenfold smaller than after separate treatments with ampicillin and ofloxacin. This suggests that 90 % of the shift-induced ampicillin and ofloxacin persisters were tolerant to only one antibiotic and not the other (Amato and Brynildsen 2015).

## Can the persister state be induced?

It is often stated that persisters are pre-existing in bacterial cultures before the antibiotic treatment (Balaban et al. 2004; Maisonneuve et al. 2013), but does this hold for all antibiotic classes and can persisters be induced? Generally, all stresses that temporarily inhibit growth seem to induce persisters. The diverse stress signals that induce persisters include metabolic limitations (i.e., secession of growth, reaching a stationary phase) (Jõers et al. 2010; Luidalepp et al. 2011), nutritional shifts/diauxie (Amato and Brynildsen 2014, 2015; Amato et al. 2013; Kotte et al. 2014), expression of toxic proteins (Vázquez-Laslop et al. 2006), and low concentrations of antibiotics (Dörr et al. 2010; Johnson and Levin 2013; Ocampo et al. 2014). Again, different effects of different antibiotics are seen; for example, ampicillin seems not to induce dormancy, whereas fluoroquinolones and aminoglycosides do (Johnson and Levin 2013; Dörr et al. 2009,2010). Induction of persisters by antibiotics raises concerns about pharmacokinetics during an antibiotic treatment regime. It is unavoidable that lower antibiotic concentrations precede the peak concentration at the infection site. Moreover, selection consisting of several rounds of antibiotic treatment resembling the setup of the Moyed’s pioneering work of *hip* mutant isolation (Moyed and Bertrand 1983) is constantly occurring in every chronic infection treated with antibiotics. Additionally, host factors, e.g., macrophage phagocytosis (Helaine et al. 2014) and location in low-pH compartments (Leimer et al. 2016) constitute harsh stressors that induce persisters during infection. Indeed, persisters strike back exactly where we most want to attack them.

## The many roads to persistence—persister formation as a function of experimental conditions

Killing by antibiotics and growth resumption are strongly influenced by growth conditions, e.g., different growth media lead to very different persister levels (Luidalepp et al. 2011; Varik et al. 2016). We have switched from autoclaving to filtration as a way of sterilization of rich media because even minor changes in the autoclaving conditions influence degradation of the ingredients and our experimental results (Luidalepp et al. 2011; Madar et al. 2013). Since growth rates are necessary to antibiotic sensitivity of the culture, it is necessary to report the growth curves alongside the persister measurements. In a recent opinion paper, Frederick Neidhardt urged for standardization and extensive documentation of the growth conditions used in microbiological studies (Neidhardt 2006); we strongly support this recommendation.

## Persister formation as a function of strain background

What is true for *E. coli* is true for the elephant—unless we study the trunk. Research on experimentally amenable model organisms is convenient as long as we can extrapolate from model organisms. Laboratory strains of *E. coli*, being the most common models in molecular microbiology, are also the bacteria of choice in investigations on persistence.

As laboratory strains of *E. coli* have preserved the key components of replication, gene expression, and metabolism, they naturally have some relevance to biological phenomena and treatment of infectious diseases. However, the key question is, do laboratory *E. coli* preserve those features of dormancy and growth resumption that are specifically relevant for persister formation?

First, we need to know the variability in natural *E. coli* isolates. Toxin-antitoxin systems are implicated in persister formation (Keren et al. 2004b; Maisonneuve et al. 2011; Gerdes and Maisonneuve 2012; Maisonneuve et al. 2013; Helaine et al. 2014), but they are components of the variable part of genome. For example, *E. coli* laboratory K12 and uropathogenic CFT073 were reported to have only a small overlap in their complement of toxin-antitoxin genes (Pandey and Gerdes 2005; Norton and Mulvey 2012). The high variability of toxin-antitoxin systems between strains was confirmed when it was shown that even the K-12 strains can contain between 9 and 16 systems (Makarova et al. 2009). Although the numbers of annotated toxin-antitoxin systems have increased over the years, the wider view of high variability is unchanged. On top of the natural variation, laboratory *E. coli* has been cultivated through numerous growth resumption cycles that might have strongly selected for specific growth resumption phenotypes (Lenski et al. 1998). Just how much the laboratory model strains reflect the persisters observed in clinical setting needs investigating.

The next layer of complications is related to the construction of knockout strains; complementation of knockouts from plasmids is the method of choice during strain construction. This practice is now not widely used as high-throughput methods emerged. Large collections of knockout strains and gene inactivation libraries are screened for mutants that have increased persister levels (Li and Zhang 2007). Similarly, these collections are often used as a source for strains for more detailed studies (Luidalepp et al. 2011; Wu et al. 2012). In case mutations influence cell growth and growth resumption parameters that are often the key issues for persister formation, compensatory mutations are selected. The procedure can result in different strains in which the inactivated genes are compensated by different mutations (Shachrai et al. 2011; Bergmiller et al. 2012). For example, Shachrai et al. (2010) have studied the *E. coli spoT* knockout strain, although this strain is not viable in the absence of compensatory mutations (Shachrai et al. 2011; Xiao et al. 1991; Montero et al. 2014). The evolution of compensatory mutations makes interpretation of the results difficult—the phenotypes measured can be caused not only by inactivation of the original gene but also by the compensatory mutations.

## A search for the universal molecular mechanism of persistence

Modern drug development starts with the identification of a target macromolecule. According to this pharmaceutical logic, targeting persisters must start with identification of their formation pathway, which would allow development of inhibitors (or activators) against the key components of the pathway. An assumption here is that a single or a few pathways exist.

The standard method of identifying such a pathway is a lack-of-function screen of a mutant library. Despite many attempts (De Groote et al. 2009; Hansen et al. 2008; Shan et al. 2015), these screens failed to identify a mutant completely lacking persisters, which would otherwise be comparable to asporogenic mutants of endospore-forming bacteria. Altogether, genetic studies have not pinpointed specialized persister genes with no role outside persister formation, suggesting a lack of a specialized persister formation pathway. The identified genes are often pleiotropic, e.g., *relA*, *spoT*, *dksA*, *ssrA*, *lon*, having multiple effects on many different biological traits. Other genes emerging from the screens are those involved in metabolism (e.g., *sucB*, *glpD*, *ubiF*) or in well-defined stress responses (e.g., *recA*), and their effects on persister formation are clearly auxiliary. Follow-up studies based on the findings of the screens show that the effects of mutations implicated in altered persistence heavily depend on the particular experimental conditions (Luidalepp et al. 2011). Because many mutations reduce to some extent persister levels, combining several knockouts to obtain larger effects looks tempting, which also allows seeing the effects of redundant genes—as shown for the TA systems (Maisonneuve et al. 2011; Maisonneuve et al. 2013). One difficulty may be the deviant physiology of the multiple knockout strains; for example, the Δ*relA*, Δ*spoT* strains have been used to test the effect ppGpp on persister formation in both *E. coli* (Maisonneuve et al. 2013) and *Pseudomonas aeruginosa* (Nguyen et al. 2011), while it is known that such double mutants result in pronounced growth defects and that they swiftly pick up compensatory mutations (Montero et al. 2014).

An important question is whether the findings illuminate universal molecular mechanisms or are restricted to specific bacterial strains and experimental setups. The role of reactive oxygen species has been suggested to be a general bactericidal mechanism of antibiotics (Kohanski et al. 2007). Although the experimental data were correct under the precise conditions used, even slight changes in experimental conditions led to data that undermined the general validity of the concept (Keren et al. 2013; Liu and Imlay 2013). A very specific model for persister formation that involves the signal molecule ppGpp and toxin-antitoxin systems has been proposed for *E. coli* by Maisonneuve et al. (2013). The general validity of this model remains to be tested in different growth conditions, and strains before it might be approved as an overarching concept. For example, persisters are still formed in *E. coli* strains lacking enzymes for ppGpp synthesis (Chowdhury et al. 2016). In addition, a mechanism that does not rely on toxin-antitoxin systems but senses the intracellular ATP level has been suggested as a key component for persister formation in *Staphylococcus aureus* (Conlon et al. 2016).

Another direction in the search for genetic determinants is screening for genes and mutants that increase persister levels, i.e., a gain-of-function screen (Girgis et al. 2012; Spoering et al. 2006). Both overexpression (Spoering et al. 2006) and disruption (Girgis et al. 2012) of many genes can considerably increase persister formation, and persistence is accompanied by a drop in growth rate. It is a matter of contention whether bacteria that have been turned into “model persisters” by artificial overexpression of growth-arresting proteins (Mok et al. 2015; Rotem et al. 2010) are suitable for studying persister physiology and metabolism.

When Moyed and Bertrand (1983) selected for mutants with altered persister levels, they tried to exclude indirect effects of impaired growth by setting requirements for unchanged growth parameters and MIC, thereby attempting to select for mutations as specific as possible for a changed persister level. This was forgotten in later screens and characterization of natural/clinical isolates, making any interpretation difficult.

## Bet hedging or just damage?

Several bacterial groups form spores, a strategy that evolved to encounter harmful environmental conditions. It is suggested that formation of the dormant persisters is also a purposeful strategy that evolved by natural selection similarly to such features as lactose metabolism or the formation of flagella. Stochastic entry of a subset of cells into dormancy and the spreading with time of their subsequent wake-up might be a bet-hedging strategy that allows cells to survive in fluctuating environments (Grimbergen et al. 2015). Similar patterns of a signal-induced fall into dormancy (inability to initiate growth under favorable conditions) and spread of reactivation times occur across many life forms. These have been described in plant seed dormancy and germination (Bentsink and Koornneef 2008), dormancy and sprouting of vegetative underground parts in plant (Sarath et al. 2014), as well as in the heterogeneity of bacterial sporulation (González-Pastor 2011; Verplaetse et al. 2015) and spore germination (Sturm and Dworkin 2015). However, it has to be noted that claiming a phenomenon “bet hedging” requires careful consideration (de Jong et al. 2011). As a fashionable concept, it is often used as a conceptual cornerstone (e.g. Verstraeten et al. 2015), although it is extremely difficult to prove that the phenomena had evolved for hedging bets.

An alternative explanation is the *P*ersistence *a*s *S*tuff *H*appens (PaSH) hypothesis advanced by Bruce Levin (Johnson and Levin 2013; Levin et al. 2014), similar to the “just damage” mechanism of Nyström and Gustavsson (1998). These concepts postulate that many mechanisms are responsible for persistence, just as there are many mechanisms in mutagenesis. Mutagenesis, damage, and aging cannot be switched off. Imagine a town attacked by deadly radiation that kills all the people not in their homes on the day of irradiation. The vast majority of townspeople to survive would be the sick and old who remained in bed. According to this model, making bacteria sick would increase persister levels, which is what happens. As already discussed, persister levels are increased by harmful environmental conditions and many genetic defects.

Nevertheless, in many circumstances, the persisters have a clear adaptive value for a bacterial population (Van den Bergh et al. 2016), for example, by allowing to establish chronic infections. Increase in persister levels of *P. aeruginosa* clinical isolates from cystic fibrosis patients implies that *hip* mutants are selected in chronic patients (Mulcahy et al. 2010); similar observation was made for the isolates of pathogenic yeasts (Lafleur et al. 2010) and clinical isolates of *S. aureus* (Gao et al. 2010) and *E. coli* (Schumacher et al. 2015). It seems that both the adaptation and the PaSH mechanisms are used by different strains and under different conditions, and these may even sometimes operate in parallel.

## Perspectives

In accepting the PaSH hypothesis and that we cannot switch off persister formation pharmaceutically, how should persister research and the development of anti-persister therapy proceed? In the light of recent research on human microbiota that shows its strong impact on the immune system and health in general, it would be unreasonable to conceive of drugs that indiscriminately kill bugs and leave a patient germ free. Rather, persisters of our healthy microbiota are extremely valuable for restoring it after an antibiotic regime. Therefore, development of drugs and treatments that specifically target particular chronic infections (sensitive bacteria + persisters) seems to be the most promising approach. There are already some preclinical examples of such approach. In a recent study, Conlon et al. recognized the unique ability of ADEP (acyldepsipeptide) to target a stationary population of *S. aureus* that was insensitive to a range of antibiotics (Conlon et al. 2013). ADEP activates and dysregulates protease ClpP by opening up the proteolytic interior of the enzyme. Development of the drug was not pursued because bacteria acquire resistance at a high frequency (1 × 10^−6^) to the compound due to null mutation of the non-essential clpP gene. However, ADEP in combination with rifampicin eradicated the population and could sterilize a deep-seated murine thigh infection model of *S. aureus*.

More research of the mechanisms underlying persisters is clearly needed, which would be expected to lead to improved antibiotics and treatment regimes. Two important points need to be emphasized: (i) the phenomenon should not be oversimplified, we should not hurry into the molecular mechanisms before the empirical aspects of persistence are described and model systems validated and, therefore, (ii) further research on animal infection models is required as only this can indicate which in vitro observations are relevant regarding any possible (clinical) therapeutic measure.
